# Porcine Sapelovirus 3C^pro^ Inhibits the Production of Type I Interferon

**DOI:** 10.3389/fcimb.2022.852473

**Published:** 2022-06-15

**Authors:** Mengge Yin, Wei Wen, Haoyuan Wang, Qiongqiong Zhao, Hechao Zhu, Huanchun Chen, Xiangmin Li, Ping Qian

**Affiliations:** ^1^ State Key Laboratory of Agricultural Microbiology, College of Veterinary Medicine, Huazhong Agricultural University, Wuhan, China; ^2^ Key Laboratory of Preventive Veterinary Medicine in Hubei Province, The Cooperative Innovation Center for Sustainable Pig Production, Wuhan, China; ^3^ Key Laboratory of Development of Veterinary Diagnostic ProductsAgriculture of the People’s Republic of China, Ministry of Agriculture of the People’s Republic of China, Wuhan, China; ^4^ International Research Center for Animal DiseaseTechnology of the People’s Republic of China, Ministry of Science and Technology of the People’s Republic of China, Wuhan, China

**Keywords:** porcine sapelovirus, 3C protease, MAVS, TBK1, MDA5

## Abstract

Porcine sapelovirus (PSV) is the causative pathogen of reproductive obstacles, acute diarrhea, respiratory distress, or severe polioencephalomyelitis in swine. Nevertheless, the pathogenicity and pathogenic mechanism of PSV infection are not fully understood, which hinders disease prevention and control. In this study, we found that PSV was sensitive to type I interferon (IFN-β). However, PSV could not activate the IFN-β promoter and induce IFN-β mRNA expression, indicating that PSV has evolved an effective mechanism to block IFN-β production. Further study showed that PSV inhibited the production of IFN-β by cleaving mitochondrial antiviral signaling (MAVS) and degrading melanoma differentiation-associated gene 5 (MDA5) and TANK-binding kinase 1 (TBK1) through viral 3C^pro^. In addition, our study demonstrated that PSV 3C^pro^ degrades MDA5 and TBK1 through its protease activity and cleaves MAVS through the caspase pathway. Collectively, our results revealed that PSV inhibits the production of type I interferon to escape host antiviral immunity through cleaving and degrading the adaptor molecules.

## Introduction

Porcine sapelovirus (PSV) is a non-enveloped positive single-stranded RNA virus, belonging to the *Sapelovirus* genus within the Picornaviridae family (Adams et al., 2015). The genome of PSV is similar to other picornaviruses: 5′-UTR-L-VP4-VP2-VP3-VP1-2A-2B-2C-3A-3B-3C-3D-3′-UTR (Krumbholz et al., 2002). Since 1960, PSV infection was reported in the United Kingdom, and it has been reported in other countries around the world, including China, South Korea, and Brazil (Donin et al., 2014; Schock et al., 2014; Son et al., 2014; Li et al., 2019). PSV infection can cause acute diarrhea, poliomyelitis, pneumonia, reproductive disorders, and other clinical symptoms (Lan et al., 2011). The infection of PSV has broad cell tropism *in vitro* (Li et al., 2019). The molecular mechanism of PSV evading host antiviral innate immunity is still unclear, and the related research contributes to revealing the pathogenicity of PSV infection and the formulation of PSV-preventive measures.

Innate immunity plays an essential role in host defense-invading pathogens (Akira et al., 2006). The host pattern recognition receptors (PRRs) can activate innate immune responses and recognize pathogen-associated molecular patterns (PAMPs) that its molecular structures were conserved within most of the pathogens (Kawai and Akira, 2006). Pathogen-associated molecular patterns (PAMPs) are mainly recognized by two types of pattern recognition receptors (PRRs), namely, the Toll-like receptor (TLR) family and retinoic acid-inducible gene-I-like receptors (RLRs) (Janeway and Medzhitov, 2002; Akira et al., 2006; Stetson and Medzhitov, 2006). During viral infection, the pathogen was captured by host pattern recognition receptor (PRR) ligands and subsequently induced IFN production mediation (Schoggins and Rice, 2011). Picornaviruses are mainly sensed by melanoma differentiation-related gene 5 (MDA5) (Gitlin et al., 2006; Kato et al., 2006; Hüsser et al., 2011)/retinoic acid-inducible gene-I-like (RIG-I) (Papon et al., 2009). These two proteins contain two caspase recruitment domains (CARD) at the N-terminus, a regulatory/inhibitory domain at the C-terminus, and a central DExD/H box ATPase/helicase. After the cytoplasmic viral RNA is detected, RIG-I and/or MDA5 will interact with mitochondrial antiviral-signaling protein (MAVS, also known as IPS-1/VISA/Cardiff) (Kawai et al., 2005; Liang-Guo et al., 2005; Meylan et al., 2005). Then, the nuclear transcription factor kappa B (NF-κB) essential modulator (NEMO) and inhibitors of κB kinase (IKK)-related kinases, such as IKK-α, -β, and -ϵ, as well as TANK-binding kinase 1 (TBK1) which leads to the various latent transcription factors were activated. These transcription factors, including interferon regulatory factor 3 (IRF-3) and NF-κB, migrate into the nucleus and directly activate promoters of type I IFNs and inflammatory cytokines/chemokines (Yoneyama and Fujita, 2007; Hiroki et al., 2011). Another family of PRRs is TLRs, in which TLR3 and TLR7/8 are known to play important roles in viral recognition (Goubau et al., 2013). TLR3 can recognize viral double-stranded RNA (dsRNA). Once activated, TLR3 recruits TIR domain-containing adaptor-inducing IFN-beta (TRIF) and activates the IKK-related kinases, including TANK-binding kinase 1 (TBK1) and inducible Iκ-B kinase (IKKi). The IKK-related kinases phosphorylate interferon regulatory factor 3/7 (IRF3/7) (Fitzgerald et al., 2003; Sharma et al., 2003; Yamamoto et al., 2003; Häcker et al., 2006; Oganesyan et al., 2006), subsequently leading to the expression of IFN-α/β (Peters et al., 2002; Hiroyuki et al., 2003; McWhirter et al., 2004).

Picornaviruses have evolved strategies to counteract the host innate immune systems. Most members of the Picornaviridae family can evade the host innate immune response by inhibiting the production of type I interferon, thus successfully proliferating and causing infection and pathogenicity. As a member of the Picornaviridae family, the molecular mechanism of PSV evading the host antiviral innate immunity is still unclear. In this study, we took the clinical isolation of PSV strains in our laboratory as the research object, and the association between PSV and the host IFN antiviral responses was investigated. Our study indicates that PSV infection cannot induce the production of type I interferon and it is an interferon-sensitive virus. In addition, PSV 3C^pro^ inhibits IFN production by degrading TBK1/MDA5 and cleaving MAVS/TANK.

## Materials and Methods

### Cells, Viruses, and Chemicals

Baby hamster kidney BHK-21 cells (BHK-21; ATCC, CCL-10), human embryonic kidney HEK293T cells (HEK293T; ATCC, CRL-11268), and porcine kidney (PK-15; ATCC, CCL-33) cells were grown in Dulbecco’s modified essential medium (DMEM; Invitrogen, Carlsbad, CA, USA) containing 10% fetal bovine serum (Gibco, Grand Island, NY, USA), 100 U/ml penicillin (Glenview, USA), and 10 μg/ml streptomycin sulfate (Glenview, USA) at 37°C in a humidified 5% CO_2_ incubator. PSV and VSV were used in this study, and virus titers were determined using plaque-forming unit assays in BHK-21 cells. Sev was amplified in chicken eggs, and viral titer was determined using the hemagglutination (HA) test. IFN-β (Sino Biological, Shanghai, China) was used for IFN stimulation. The proteasome inhibitor MG132 (S1748-1mg), the caspase inhibitor zVAD-FMK (C1202), dimethyl sulfoxide (DMSO, ST038), and NH4Cl were obtained from Beyotime Biotechnology.

### Antibody

The commercial antibodies used in this study including anti-FLAG monoclonal antibody and anti-HA monoclonal antibody were obtained from Medical and Biological Laboratories (Nagoya, Japan); anti-IRF3 polyclonal antibody (11312-1-AP) and anti-TANK polyclonal antibody (27065-1-AP) were obtained from Proteintech (Wuhan, China); anti-p-IRF3-386 (AP0995) monoclonal antibody, anti-TBK1 monoclonal antibody (A14641), anti-GAPDH (AC002) monoclonal antibodies, and anti-β-tubulin monoclonal antibodies (AC012) were obtained from ABclonal (Woburn, MA, USA); anti-MAVS monoclonal antibody (SC-166583) was obtained from Santa Cruz Biotechnology (Dallas, TX, USA); anti-MDA5 polyclonal antibody (M00263) was obtained from Boster (Pleasanton, CA, USA); anti-PARP1 polyclonal antibody (GB111500) was obtained from Servicebio (Wuhan, China); and anti-PSV VP1 polyclonal antibody was prepared in our laboratory.

### Plasmids

The full-length CDs of individual PSV viral proteins were amplified from PSV (MT080999.1) cDNA. VP1, VP2, VP3, 3C^pro^, and 3D were cloned into vector pCAGGS-HA. L, 2A, 2C, and 3A were cloned into pEBG-GST. VP4 was cloned into vector pEGFP-C1, and 2B was cloned into vector pCDNA3.1-Flag. Flag-TANK was a kind gift from Changjiang Weng (Harbin Veterinary Research Institute of Chinese Academy of Agricultural Sciences). Flag-MAVS and Flag-TRIF were kindly provided by Mei lin Jin (Hua Zhong Agricultural University). Mutagenesis of PSV 3C^pro^, MAVS, and TANK constructs was performed using overlap PCR (C214-01/02, Vazyme, Nanjing, China). All constructs were identified through DNA sequencing. Primer pairs used in this study are shown in Supporting Table 1. Mutants of human MAVS (D429A, D490A, and D498A) and PSV 3C^pro^ (H40A, C146A, and H40A/C146A) were constructed by overlap extension using Pfu DNA polymerase (Stratagene, La Jolla, CA). The cDNAs encoding deletion mutants of MAVS, including N429 (1–429 amino acids) and C430 (430–540 amino acids), were cloned into the pFlag vector.

### Western Blotting

Total cellular proteins were prepared using lysis buffer (1.19% HEPES, 0.88% NaCl, 0.04% EDTA, 1% NP-40, and a protease inhibitor) with occasional vortexing. Lysates were then collected by centrifugation at 12,000 rpm for 10 min at 4°C, and protein concentrations were determined by the bicinchoninic acid protein assay kit (Thermo Scientific, Waltham, MA, USA). The equal amounts of protein for each sample were loaded and separated by 8% to 12% SDS-PAGE and transferred onto polyvinylidene fluoride (PVDF) membranes (Roche, Welwyn Garden City, UK). Membranes were blocked with 5% skim milk in PBST with 5% Tween 20 (DGBio, Beijing, China) for 2 h at room temperature. The membranes were then washed two times with PBST and incubated with primary antibodies at room temperature for 2 h. Afterward, the membranes were washed five times with PBST and incubated with anti-rabbit or anti-mouse IgG antibodies conjugated to horseradish peroxidase (HRP) at room temperature for 1 h. The membranes were developed using enhanced chemiluminescence detection reagents (Thermo Scientific, USA).

### Luciferase Reporter Gene Assay

HEK293T cells were seeded in 24-well plates, and the monolayer cells were co-transfected with 100 ng/well of pGL3-IFNβ-Luc plasmid, 5 ng/well of pRL-TK plasmid (as an internal control), and the indicated expression plasmids or an empty control plasmid. The cells were infected PSV or Sev 20 h after the initial co-transfection. The cells were then collected and lysed, and firefly luciferase and Renilla luciferase activities were determined using the dual-luciferase reporter assay system (Beyotime, Shanghai, China) according to the manufacturer’s protocol. Three independent experiments were performed in duplicate. Data are presented as means ± standard deviations(SD).

### Immunofluorescence

Cells were fixed in 4% paraformaldehyde at 4°C for 10 min and permeabilized with 0.1% Triton X-100 at room temperature for 10 min; the cells were incubated with 2% BSA in PBS for 1 h at room temperature. The cells were then washed three times with PBS and incubated with anti-IRF3 for 2 h. After four washes with PBS, the cells were incubated with Alexa Fluor 488-conjugated goat anti-rabbit IgG antibodies at room temperature for 1 h. The cells were washed again four times with PBS and then incubated with DAPI (Sigma, St. Louis, MO, USA) for 10 min, followed by five washes. Fluorescent images were examined using inverted fluorescence.

### Real-Time RT-PCR

Total RNA was extracted from the targeted cells using TRIzol reagent (Invitrogen) in accordance with the manufacturer’s instructions. 1 µg total RNA was reverse transcribed to cDNA using HiScript III 1st Strand cDNA Synthesis Kit (Vazyme). The relative mRNA level of targeted genes was measured with qPCR using SYBR Green real-time PCR master mix (Applied Biological Materials Inc) with specific prime. All reactions were performed in triplicate. The mRNA level of housekeeping gene GAPDH was used as an internal control. The relative gene fold was determined with the comparative cycle threshold (2^-ΔΔCT^) method.

### Statistical Analysis

Statistical analysis was conducted using GraphPad Prism software, version 5. All results were determined by at least three times independent experiments. The various treatments were compared using an unpaired, two-tailed Student t-test with an assumption of unequal variance. *P* value < 0.05 was considered statistically significant.

## Result

### PSV Infection Does Not Induce Type I Interferon Production

**Figure 1:**
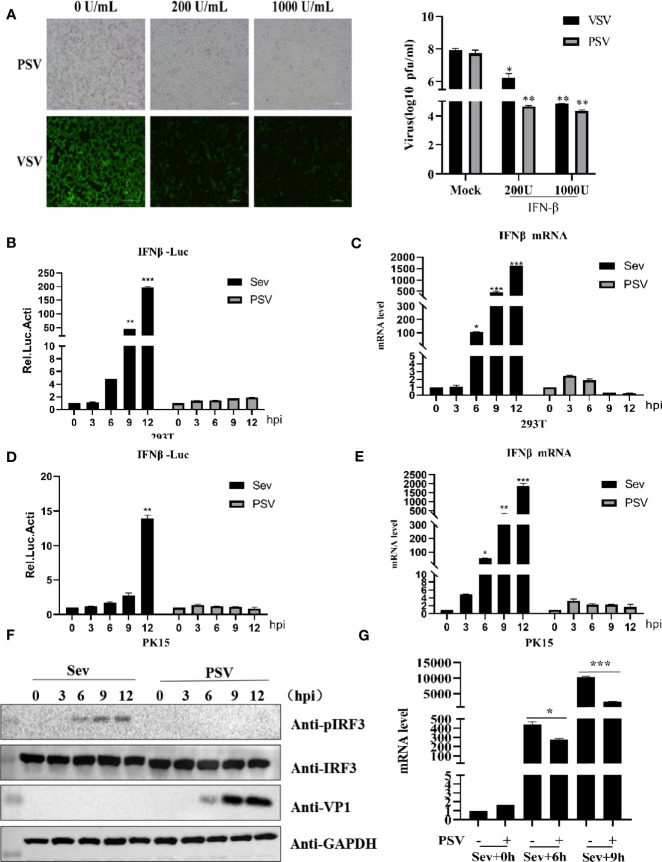
PSV infection inhibits host type I IFN production. **(A)** HEK293T cells were seeded in 12-well plates, and the monolayer cells were pretreated with IFN-β at the indicated concentration for 12 h. Then the cells were infected with VSV-GFP (0.1 MOI) or SVV (0.01 MOI) for 24 h. The CPE was observed in microscopy. Virus titer was determined by plaque assay. **(B)** HEK293T cells were seeded in twenty-four-well plates, and the monolayer cells were co-transfected with 100 ng IFN-β reporter and 5 ng pRL-TK (as an internal control) plasmids for 20 h. Then the cells were infected with Sev (25 HA) or PSV (0.1 MOI) or not for the indicated times. The cells were then subjected to dual-luciferase assays at 12 h postinfection (hpi). **(C)** HEK293T cells were seeded in 24-well plates, and the monolayer cells were infected with Sev (25 HA) or PSV (0.1 MOI) or not for the indicated times. The mRNA expression level of IFN-β was evaluated with qPCR assay, and housekeeping gene GAPDH was used as the control. **(D)** PK15 cells were seeded in 24-well plates, and the monolayer cells were co-transfected with 200 ng IFN-β reporter and 5 ng pRL-TK (as an internal control) plasmids for 20 h. Then the cells were infected with Sev (25 HA) or PSV (0.1 MOI) or not for the indicated times. The cells were then subjected to dual-luciferase assays at 12 h postinfection (hpi). **(E)** PK15 cells were seeded in 24-well plates, and the monolayer cells were infected with Sev (25 HA) or PSV (0.1 MOI) or not for the indicated times. The mRNA expression level of IFN-β was evaluated with qPCR assays, and housekeeping gene GAPDH was used as the control. **(F)** HEK293T cells were seeded in 24-well plates, and the monolayer cells were infected with Sev (25 HA) or PSV (0.1 MOI) or not for the indicated times. The cells were collected for Western blotting. **(G)** HEK293T cells were seeded in 12-well plates, and the monolayer cells were infected with PSV (0.5 MOI) or not for 3 h, then infected or not with Sev (25 HA) for another 6 and 9 h. The mRNA expression level of IFN-β was evaluated with qPCR assays, and housekeeping gene GAPDH was used as the control. Data are represented. Student’s t-test: *P < 0.05, **P < 0.01, ***P < 0.001.

Previous studies have reported that IFN-β can inhibit several picornavirus replication, such as FMDV, EMCV, and SVV (Chinsangaram et al., 2001; Wang et al., 2011; Qian et al., 2017). To evaluate whether IFN-β could inhibit PSV proliferation, HEK293T cells were pretreated with different doses of IFN-β for 12 h and then infected with PSV (0.01 MOI) for 24 h. We found that IFN-β inhibited PSV-induced cytopathic effects (CPE) and PSV proliferation in a dose-dependent manner **(** **Figure 1A** **)**. Taken together, our results demonstrate that PSV is an interferon-sensitive virus. The impact of PSV infection on the host innate immune response is unknown. The effects of PSV infection on IFN-β promoter activity were analyzed by dual-luciferase assays. We found that IFN-β promoter activity was hardly activated in PSV-infected cells **(** **Figure 1B** **)**. HEK293T cells were infected with PSV (0.1 MOI) or Sev (2^5^ HA) for the indicated time, and the expression level of IFN-β mRNA was detected through quantitative real-time RT-PCR assay. Interestingly, the mRNA level of IFN-β hardly increased in PSV-infected cells as the infected time increased **(** **Figure 1C** **)**. Similarly, we observed that the IFN-β promoter activity and the mRNA level of IFN-β hardly increased in PSV-infected PK15 cells **(** **Figures 1D, E** **)**. We also measured the phosphorylation level of IRF3 *via* Western blotting. HEK293T cells were infected with PSV (0.1 MOI) or Sev (2^5^ HA), and then the cells were collected at indicated times. The phosphorylation of IRF3 could not be detected **(** **Figure 1F** **)**. In addition, we also analyzed whether PSV could influence Sev-induced IFN-β production. The quantitative real-time PCR results showed that PSV infection inhibited the expression level of Sev-induced IFN-β mRNA **(** **Figure 1G** **)**. Collectively, these results indicate that PSV infection cannot induce type I interferon production.

### PSV 3C^pro^ Negatively Regulates the Production of Type I Interferon

**Figure 2:**
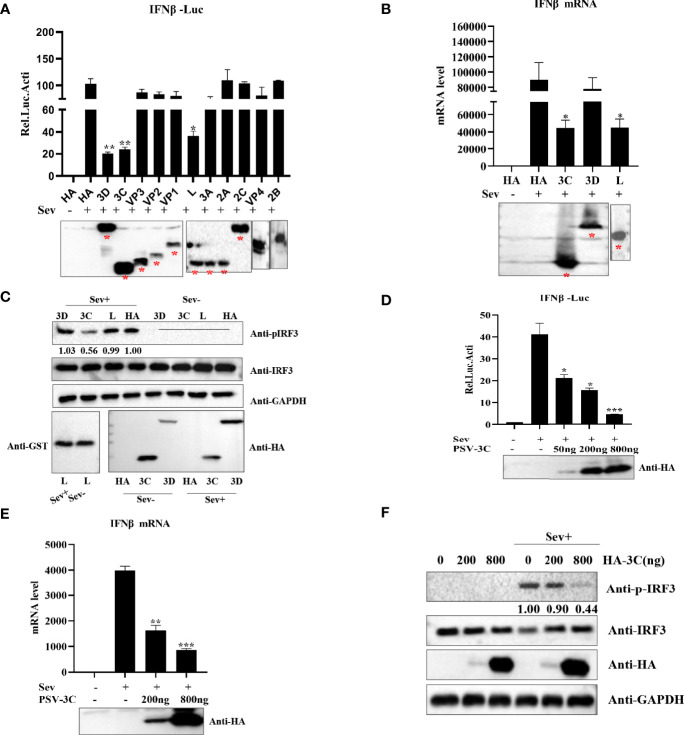
PSV 3C^pro^ suppresses Sev-induced type I IFN production. **(A)** HEK293T cells were seeded in 24-well plates, and the monolayer cells were co-transfected with 300-ng plasmids expressing indicated PSV viral protein or empty plasmids and 100 ng IFNβ-Luc along with 5 ng pRL-TK for 20 h, and then the cells were infected or uninfected with Sev (2^5^ HA) for 12 h. The cells were then subjected to dual-luciferase assays. PSV viral protein expression was detected by Western blotting. **(B)** HEK293T cells were seeded in 24-well plates, and the monolayer cells were transfected with 300-ng empty plasmids, GST-L, HA-3C, or HA-3D for 20 h, and then the cells were infected with Sev (2^5^ HA) or not. The mRNA expression level of IFN-β was measured by qPCR assay, and housekeeping gene GAPDH was used as the control. L, 3C^pro^, or 3D expression was detected by Western blotting. **(C)** HEK293T cells were seeded in 12-well plates, and the monolayer cells were transfected with 500 ng GST-L, HA-3C, and HA-3D for 20 h, and then the cells were infected with Sev (2^5^ HA) or not. The cells were collected for Western blotting. **(D)** HEK293T cells were co-transfected with different doses of 3C^pro^-expressing plasmids (0 ng, 50 ng, 200 ng, 800 ng) and 100 ng IFNβ-Luc along with 5 ng pRL-TK for 20 h. The cells were infected with Sev (2^5^ HA) or not for 12 h. The cells were then subjected to dual-luciferase assays. 3C^pro^ expression was detected by Western blotting using the HA antibody. **(E)** HEK293T cells were transfected with different doses of 3C-expressing plasmids (0 ng, 200 ng, 800 ng) for 20 h and then infected or not with Sev (2^5^ HA) for the indicated times. The mRNA expression level of IFN-β was evaluated with qPCR assays, and housekeeping gene GAPDH was used as the control. 3C^pro^ expression was detected by Western blotting using the HA antibody. **(F)** HEK293T cells were transfected with different doses of HA-3C (0 ng, 200 ng, 800 ng) for 20 h, and then the cells were infected or uninfected with Sev (2^5^ HA). The cells were collected for Western blotting. Data are represented as means ± SD. Student’s t-test: *P < 0.05, **P < 0.01, ***P < 0.001.

Based on above findings, we evaluated which viral proteins would likely be involved in the inhibition of type I production. HEK293T cells were transfected with plasmids expressing L, VP1, VP2, VP3, VP4, 2A, 2B, 2C, 3A, 3C^pro^, or 3D in combination with IFN-β-Luc and pRL-TK. The results indicated that viral proteins L, 3C^pro^, and 3D could significantly inhibit IFN-β promoter activity **(** **Figure 2A** **)**. Previous studies revealed that picornaviruses evolved various strategies to inhibit IFN-β production by certain viral proteins, such as L^pro^, VP2, VP3, 2A^pro^, 2B, 2C, 3A, 3B, and 3C^pro^ (Dang et al., 2011; Mukherjee et al., 2011; Bei et al., 2013; Dang et al., 2014; Zixiang et al., 2016; Dan et al., 2016; Dan et al., 2016; Wen et al., 2019; Xiangle et al., 2020). Our data showed that overexpression of L, 3C^pro^, and 3D reduced Sev-induced IFN-β production. Moreover, the results of quantitative real-time PCR and Western blotting demonstrated that 3C^pro^ exhibited an extreme inhibitory effect on IFN-β production **(** **Figures 2B, C** **)**. This implies that 3C^pro^ is an antagonist protein to inhibit IFN-β production. HEK293T cells were transfected with an increasing dose of HA-3C for 24 h. As shown in **Figure 2D**, PSV 3C^pro^ could significantly inhibit Sev-induced IFN-β promoter activity. In addition, PSV 3C^pro^ decreased Sev-induced endogenous transcription of IFN-β in a dose-dependent manner **(** **Figure 2E** **)**. Furthermore, we also found that the phosphorylation of IRF3 gradually reduced when the protein level of PSV 3C^pro^ was increased **(** **Figure 2F** **)**. Taken together, these data confirm that PSV 3C^pro^ inhibits type I IFN production in a dose-dependent manner.

### PSV 3C^pro^ Suppresses Sev-Induced Type I IFN Production Through Its Protease Activity

**Figure 3:**
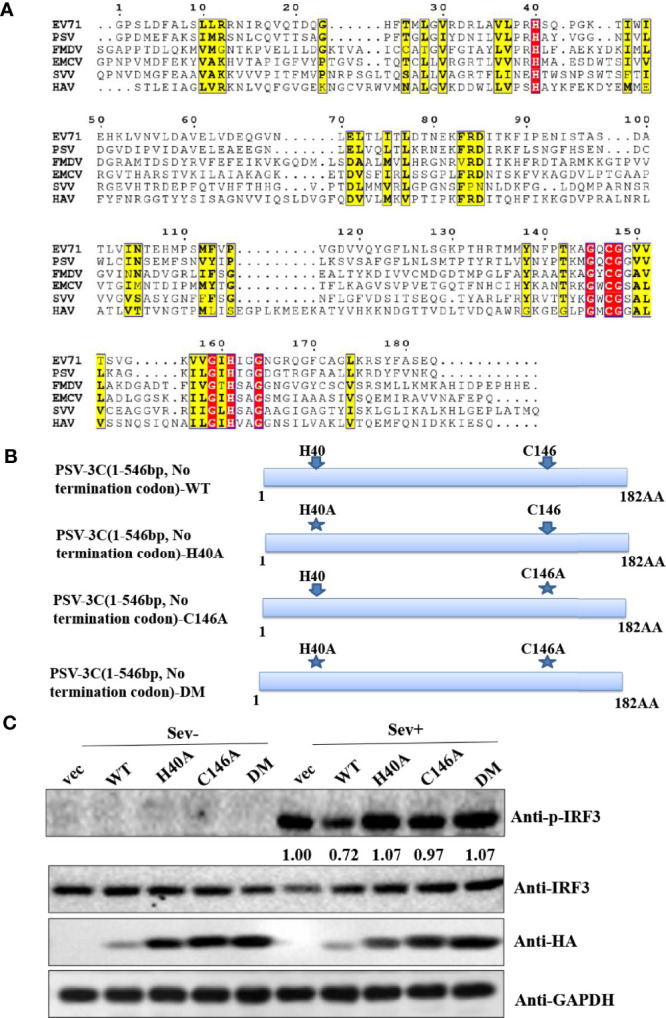
PSV 3C^pro^ suppresses Sev-induced type I IFN production dependent on its protease activity of 3C. **(A)** Amino acid sequence alignment results of 3C^pro^ derived from PSV (MT080999), SVV (APA28975), FMDV (NP74046), EMCV (NP740410), HAV (AKI05745), and EV71 (AFP66570). **(B)** Schematic diagram of PSV 3C^pro^ and its mutants. **(C)** HEK293T cells were transfected with 500 ng HA-3C or its mutants for 20 h, and then the cells were infected or not with Sev (2^5^ HA) for 9 h. The cells were collected for Western blotting.

Similar to other picornaviruses 3C^pro^, PSV 3C^pro^ contains the conserved catalytic box with histidine (His) and cysteine (Cys) residues **(** **Figure 3A** **)**. Therefore, we constructed a series of PSV 3C^pro^ mutation expression plasmids, including single-site mutations H40A and C146A and double-site mutation H40A-C146A (3CDM) **(** **Figure 3B** **)**. The result showed that wild-type 3C^pro^ (WT) suppressed Sev-induced phosphorylation of IRF3. In contrast, all 3C^pro^ mutants [3C-H40A, 3C-C146A, and H40A-C146A (3C-DM)] lost the ability to inhibit the phosphorylation of IRF3 **(** **Figure 3C** **)**. This result suggested that 3C^pro^ inhibited Sev-induced type I interferon production *via* its protease activity.

### PSV 3C^pro^ Inhibits IFN-β Expression by Targeting the Adaptor MAVS, MDA5, and TBK1

**Figure 4:**
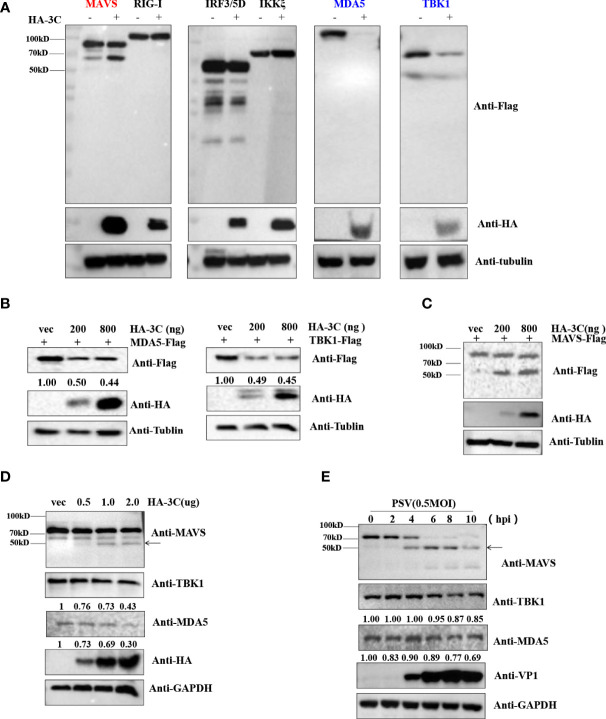
PSV 3C^pro^ inhibits IFN-β activation by downregulation of MDA5/TBK1 and cleavage of MAVS. **(A)** HEK293T cells were seeded in 24-well plates, and the monolayer cells were co-transfected with 300 ng indicated adaptor-expressing plasmids and 300 ng of empty vector or a plasmid encoding HA-3C for 24 h. The cells were collected for Western blotting. **(B,C)** HEK293T cells were co-transfected with different doses of HA-3C (0 ng, 200 ng, 800 ng), and 500 ng indicated adaptor-expressing plasmids. The cells were collected for Western blotting. **(D)** HEK293T cells were transfected with different doses of HA-3C (0.5 μg, 1 μg, 2 μg) for 24 h. The cells were collected for Western blotting. **(E)** HEK293T cells were infected with 0.5 MOI PSV for the indicated times, and then the cells were collected for Western blotting. Data are represented as means ± SD.

Previous studies demonstrated that several picornavirus 3C proteases cleave or degrade innate immune adaptors to evade host innate immune responses. SVV 3C^pro^ cleaves MAVS, TRIF, and TANK to weaken type I IFN production (Qian et al., 2017). CVB3 and EV71 cleave RIG-I *via* viral 3C^pro^ (Feng et al., 2014). SVV 3C^pro^ can target RIG-I, IRF3, and IRF7 for degradation to downregulate IFN-β production (Qiao et al., 2018; Wen et al., 2019). To investigate which innate immune adaptors are cleaved or degraded by PSV 3C^pro^, HEK293T cells were transfected with plasmids expressing FLAG-MAVS, -RIG-I, -MDA5, -IRF3, -TBK1, or -IKKξ in combination with an empty vector or plasmids expressing HA-3C. As shown in **Figure 4A**, overexpression of PSV 3C^pro^ could cleave MAVS, and we observed that TBK1 and MDA5 were degraded by PSV 3C^pro^. To confirm whether PSV 3C^pro^ degraded TBK1 and MDA5 in a dose-dependent manner, HEK293T cells were co-transfected with increasing amounts of PSV 3C^pro^ and MDA5 or TBK1 for 24 h. Western blotting results showed that the abundance of MDA5 and TBK1 was gradually reduced when the protein level of PSV 3C^pro^ was increased **(** **Figure 4B** **)**. Meanwhile, HEK293T cells were co-transfected with FLAG-MAVS and an increasing dose of PSV 3C^pro^. We observed that the cleavage products of MAVS were increased by PSV 3C^pro^ in a dose-dependent manner **(** **Figure 4C** **)**. In addition, we also assessed the effects of PSV 3C^pro^ on endogenous MDA5 and TBK1 protein expression and endogenous MAVS cleavage. HEK293T cells were transfected with increasing amounts of HA-3C for 24 h. The result showed that endogenous MDA5 and TBK1 were degraded by 3C^pro^ and 3C^pro^ cleaved endogenous MAVS **(** **Figure 4D** **)**. We also analyzed the expression of MDA5 and TBK1 and the cleavage of MAVS during PSV infection. HEK293T cells were infected with 0.5 MOI PSV for the indicated time. We found that the expression of MDA5 and TBK1 was gradually reduced during PSV infection, and PSV infection induced the cleavage of MAVS **(** **Figure 4E** **)**. Collectively, our data indicated that PSV 3C^pro^ targets MAVS, MDA5, and TBK1 to impair type I IFN production.

### PSV 3C^pro^ Reduces the mRNA Level of TBK1 and MDA5

**Figure 5:**
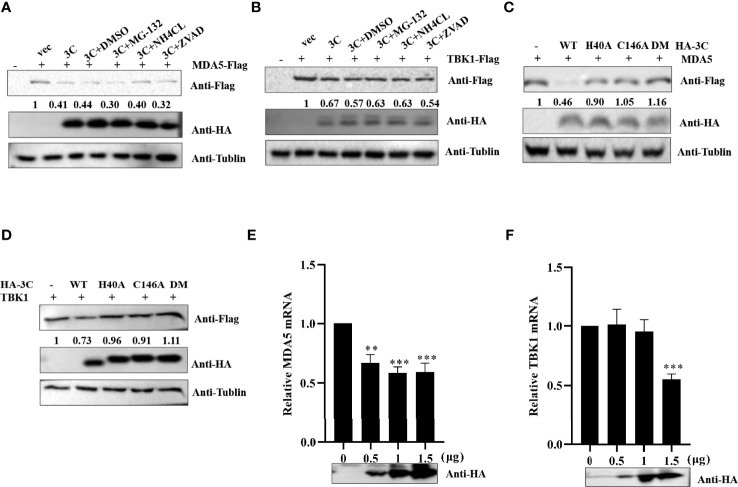
PSV 3C^pro^ inhibits the transcription level of MDA5 and TBK1. **(A,B)** HEK293T cells were seeded in 12-well plates, and the monolayer cells were co-transfected with 500 ng Flag-MDA5 or Flag-TBK1 and HA-3C for 12 h. Then the cells were treated with DMSO, MG132, NH4Cl, and Z-VAD-FMK for 12 h. The cells were collected for Western blotting. **(C,D)** The monolayer HEK293T cells in 12-well plates were co-transfected with 500 ng Flag-MDA5 or Flag-TBK1 and HA-3C or HA-3C-mutant. At 24 h post-transfection (hpt), the cells were collected and lysed for Western blotting. **(E,F)** The monolayer HEK293T cells in 12-well plates were co-transfected with different doses of HA-3C (0 ng, 200 ng, 800 ng) for 24 h, and then the cells were collected for RT-qPCR. 3C^pro^ expression was detected by Western blotting using the HA antibody. Student’s t-test: **P < 0.01, ***P < 0.001.

A previous study demonstrated that SVV 3C^pro^ degraded RIG-I through the caspase pathway (Wen et al., 2019). To investigate whether PSV 3C^pro^-mediated MDA5 and TBK1 is dependent on apoptosis-related caspase activity, proteasome, or lysosome signaling, HEK293T cells expressing MDA5 or TBK1 and PSV 3C^pro^ were treated with DMSO, proteasome inhibitor MG132 (10 μM), lysosomal inhibitor NH4Cl (10 mM), or pan-caspase inhibitor Z-VAD-FMK (50 μM). As shown in **Figures 5A, B**, we found that none of these inhibitors rescued MDA5 or TBK1 expression. We next examined whether 3C^pro^-induced reduction of MDA5 and TBK1 depended on its protease activity; HEK293T cells were co-transfected with FLAG-MDA5 or -TBK1 and all 3C^pro^ mutants (H40A, C146A, and H40A-C146A (3C-DM)) for 24 h. Western blot results showed that 3C^pro^ degraded MDA5 and TBK1 through its protease activity **(** **Figures 5C, D** **)**. Next, we tested whether PSV 3C^pro^ downregulated the level of TBK1 and MDA5 mRNA. The result of qRT-PCR suggested that PSV 3C^pro^ could significantly reduce the mRNA level of TBK1 and MDA5 **(** **Figures 5E, F** **)**.

### PSV 3C^pro^ Cleaves MAVS Through the Apoptosis Pathway

**Figure 6:**
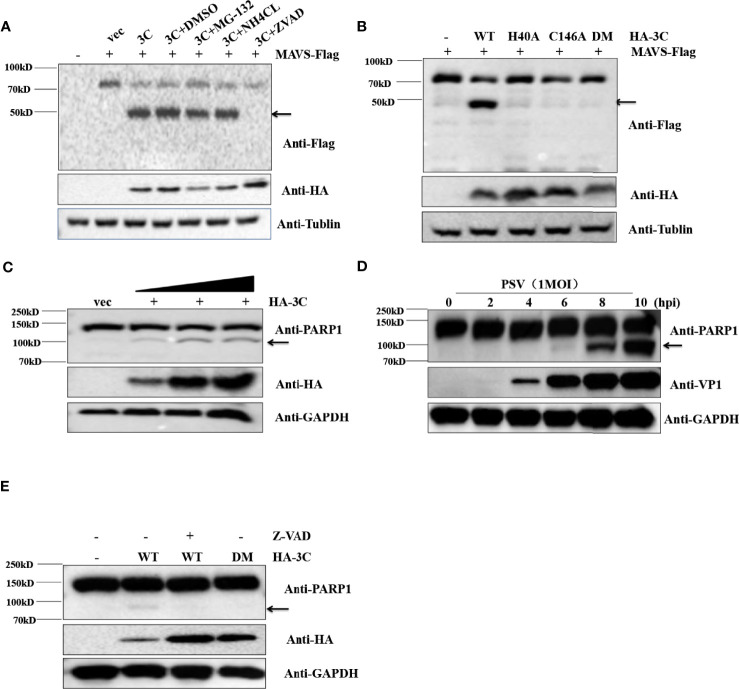
PSV 3C^pro^ cleaves MAVS through the apoptosis pathway. **(A)** HEK293T cells were seeded in 12-well plates and co-transfected with 500 ng Flag-MAVS and HA-3C for 12 h, then the cells were treated with DMSO, MG132, NH_4_Cl, and Z-VAD-FMK for 12 h. The cells were then collected for Western blotting. **(B)** The monolayer HEK293T cells in 12-well plates were co-transfected with 500 ng Flag-MAVS and HA-3C or HA-3C-mutant. At 24 hpt, the cells were collected and lysed for Western blotting. **(C)** HEK293T cells were infected with 0.5 MOI PSV for the indicated times, and then the cells were collected and lysed for Western blotting. **(D)** HEK293T cells were transfected with different doses of HA-3C (0.5 μg, 1 μg, 2 μg) for 24 h, and then the cells were collected and lysed for Western blotting. **(E)** HEK293T cells were transfected with 500 ng HA-3C or its mutant for 12 h. The Z-VAD-FMK (50 μM) were added and maintained in the cells for another 12 h, and then the cells were collected and lysed for Western blotting.

Previous reports have clearly shown that MAVS could be cleaved by virus proteases (NS3/4A and 3ABC) or caspases (Yang et al., 2007; Yu et al., 2010; Anggakusuma et al., 2016). To determine whether cellular proteases participate in 3C^pro^-mediated cleavage of MAVS, the effect of proteasome inhibitor MG132, lysosomal inhibitor NH4Cl, and pan-caspase Z-VAD-FMK on the cleavage of MAVS were detected by Western blot. Flag-MAVS and PSV 3C^pro^ were co-transfected into HEK293T cells for 12 h, and then the cells were treated with DMSO, MG132 (10 μM), NH4Cl (10 mM), and Z-VAD-FMK (50 μM) for another 12 h. The results showed that PSV 3C^pro^ lost the ability to mediate the cleavage of MAVS in the presence of Z-VAD-FMK, indicating that caspases were involved in 3C^pro^-mediated MAVS cleavage **(** **Figure 6A** **)**. To investigate whether 3C protease activity also participated in 3C^pro^-mediated cleavage of MAVS, FLAG-MAVS and 3C mutants [3C-H40A, 3C-C146A, and 3C-H40A-C146A (3C-DM)] were co-transfected into HEK293T cells for 24 h. Compared to the wild-type PSV 3C^pro^, all 3C^pro^ mutants could not cleave MAVS **(** **Figure 6B** **)**. To confirm whether PSV 3C^pro^ could induce cell apoptosis, HEK293T cells were transfected with an increased amount of HA-3C. As shown in **Figure 6C**, PSV 3C^pro^ cleaved PARP1 in a dose-dependent manner. This result revealed that 3C^pro^ could induce apoptosis. Interestingly, we found that PSV could also induce cell apoptosis **(** **Figure 6D** **)**. To determine whether 3C^pro^ induced apoptosis through its protease activity, HEK293T cells were transfected with the HA-3C or 3C^pro^ mutant. PSV 3C^pro^ lost the ability to cleave PARP1 in the presence of Z-VAD-FMK, and PSV 3C^pro^ without protease activity could not cleave PARP1 **(** **Figure 6E** **)**. These data showed that the protease activity was essential for PSV 3C^pro^-mediated apoptosis.

### PSV 3C^pro^-Mediated MAVS Cleavage Products Lose the Ability to Induce Type I Interferon Production

**Figure 7:**
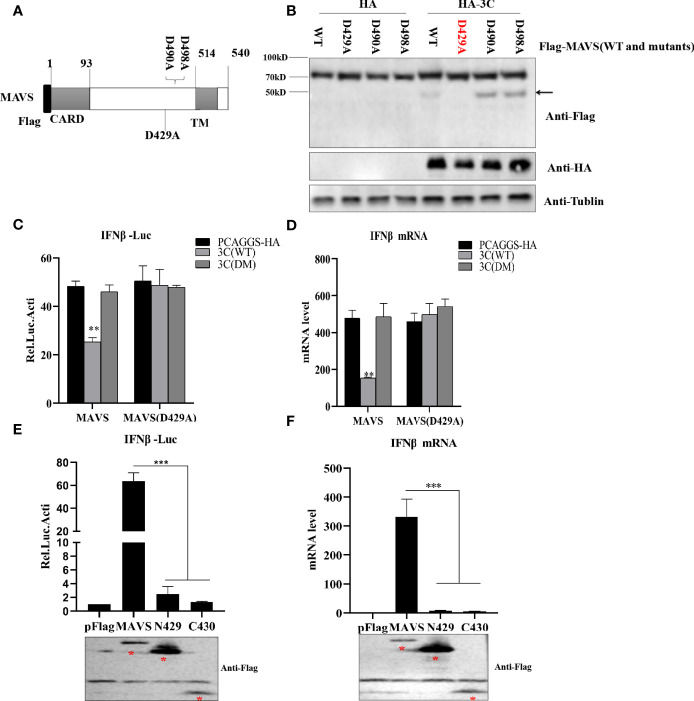
PSV 3C^pro^ cleaves MAVS at D429. **(A)** Schematic diagram of MAVS and its mutants. **(B)** HEK293T cells were co-transfected with 500 ng HA-3C and Flag-MAVS or its mutants for 24 h. Cell lysates were analyzed by Western blotting. **(C, D)** HEK293T cells were transfected with 300 ng MAVS or MAVS (D429A) and HA-3C (WT) or HA-3C (DM). Luciferase activity assay assessed IFN-β promoter activity and qPCR tested the mRNA expression level of IFNβ. **(E, F)** The effects of cleavage fragments of MAVS by PSV 3C^pro^ on MAVS-mediated IFN-β production were assessed *via* luciferase activity assay and qPCR assay. The expression of MAVS and its mutants was detected by Western blotting using the FLAG antibody. Data are represented as means ± SD. Student’s t-test: *P < 0.05, **P < 0.01, ***P < 0.001.

To investigate the 3C^pro^-mediated MAVS cleavage site, we constructed three MAVS mutants in which aspartic (D) was replaced with alanine (A) **(** **Figure 7A** **)**. HEK293T cells were transfected with MAVS or MAVS mutants and HA-3C. As shown in **Figure 7B**, we observed that the cleavage fragment entirely disappeared in MAVS-D429A. Taken together, these results demonstrated that PSV 3C^pro^ cleaved MAVS at D429. MAVS is the key adaptor molecule in RLR signaling pathways. To investigate whether PSV 3C^pro^ could disrupt MAVS-mediated type I interferon production, a dual-luciferase activity assay was performed to detect the IFN-β promoter activity. The result showed that MAVS-mediated IFN-β promoter activity was significantly inhibited by 3C-WT, but not 3C-DM **(** **Figure 7C** **)**. In addition, MAVS-D429A-mediated IFN-β promoter activity was not affected by 3C-WT or 3C-DM, suggesting that MAVS-D429A could resist 3C^pro^-mediated cleavage and retain the ability of IFN-β promoter activity **(** **Figure 7C** **)**. To confirm the results, HEK293T cells were transfected with MAVS, MAVS-D429A, and 3C-WT or 3C-DM for 24 h, and the cells were collected for qRT-PCR analysis. As expected, MAVS-D429A could induce IFN-β production and 3C-WT did not inhibit MAVS-D429A-mediated IFN-β production **(** **Figure 7D** **)**. We next assessed whether MAVS cleavage fragments maintain the activity of MAVS-mediated IFN-β production; the truncated plasmids expressing the N-terminal (residues 1 to 429; N429) and C-terminal (residues 430 to 540; C430) fragments of MAVS were constructed, and the effect was evaluated by dual-luciferase activity assay and qRT-PCR. The cleavage products cannot activate IFN-β promotor activity and increase the IFN-β mRNA level, indicating that N429 and C430 lost the ability to induce IFN-β production **(** **Figures 7E, F** **)**. Taken together, PSV 3C^pro^-mediated MAVS cleavage products lose their ability to induce type I interferon production.

## Discussion

The innate immune response is the key of host against invading pathogens, and it is the first line of host defense against pathogen invasion (Schoggins and Rice, 2011; Qiao et al., 2018). The type I interferon (IFN) family, including IFN-α and IFN-β, is an essential component of the host innate immune response and the first line of host response against invading pathogens (Sadler and Williams, 2008). Pathogen ligands interact with pattern recognition receptors (PRRs), and thereby the IFN regulators (IRFs) induce interferon (IFN) production. Subsequently, the IFN molecules bind to IFN receptors and activate numerous IFN-stimulated genes (ISGs), which directly or indirectly exert antiviral functions (Schoggins and Rice, 2011; Schneider et al., 2014). However, the host cells have evolved highly specialized mechanisms to detect and resist virus invasion. However, many viruses have developed countermeasures to disrupt these signaling pathways (Dang et al., 2012). Picornavirus mainly destroys the RLR signaling pathway to inhibit IFN production. SVV 3C^pro^ cleaves MAVS, TRIF, and TANK to abrogate host innate immune responses (Qian et al., 2017). The cleavage of TANK by EMCV 3C^pro^ impairs the ability of TANK to inhibit TRAF6-mediated NF-κB signaling, which helps it evade the host innate immune responses (Li et al., 2015). HAV cleaves MAVS to ablate RLR-mediated type I IFN response (Yang et al., 2007). Enterovirus infection induces the cleavage or degradation of host factors, including RIG-I, MDA5, MAVS, TRIF, and IRF7/9 (Xiaobo et al., 2016). FMDV infection restricts the expression of RIG-I, MDA5, and MAVS (Dan et al., 2016; Zixiang et al., 2016).

Previous studies have reported that some other picornavirus 3C^pro^ impairs type I IFN production (Dang et al., 2012; Xiaobo et al., 2016; Qian et al., 2017). In this study, we found that PSV infection could inhibit type I interferon production. PSV 3C^pro^ inhibited IFN production dependent on its protease activity. PSV 3C^pro^ suppressed type I interferon production by degrading TBK1/MDA5 and cleaving MAVS, which depends on 3C protease activity. This degradation is not associated with cellular proteasome-, lysosome-, or apoptosis-mediated degradation. Surprisingly, our study demonstrated that PSV 3C^pro^ could not interact with TBK1 and MDA5 (data not shown). Previous studies have suggested that FMDV 3A inhibited the expression of MDA5 by disrupting their mRNA level (Dan et al., 2016). Similarly, PSV 3C^pro^ could significantly reduce the mRNA levels of TBK1 and MDA5, indicating that PSV 3C^pro^ degrades TBK1 and MDA5 by targeting transcription levels. Meanwhile, we detected the endogenous degradation of TBK1/MDA5 in PSV-infected or PSV 3C^pro^-overexpressed cells. SVV 3C^pro^ cleaved MAVS depending on its protease activity (Qian et al., 2017). Interestingly, our data suggested that PSV infection and 3C^pro^ expression could induce apoptosis, which is the key to 3C^pro^-mediated MAVS cleavage. Apoptosis is a unique and important way of programmed cell death, involving genetically determined cell elimination (Fomigli et al., 2000; Sperandio et al., 2000; Martinvalet et al., 2005). Apoptosis usually occurs in the process of cell development and senescence, which maintains the stable number of cells in the tissue. In addition, apoptosis also occurs as a defense mechanism, such as in immune response or when cells are destroyed by diseases or toxic substances (Norbury and Hickson, 2001). Many viruses have developed different strategies to mediate apoptosis to ensure their continued reproduction and/or spread (Cuconati and White, 2002). SVV impacts the extrinsic and intrinsic pathway and then mediates apoptosis (Tingting et al., 2019). Enterovirus 71 (EV71) infection induces apoptosis in many cell lines through impacting the mitochondrial apoptotic pathway (Liang et al., 2004; Shih-Cheng et al., 2004). SARS-CoV ORF7a and 7b induce apoptosis by activating caspase 3 (SS et al., 2007). HIV-1 Vpr destroys mitochondrial transmembrane potential and activates both caspases-8 and 9 to induce apoptosis (Jacotot et al., 2000; Nie et al., 2002). HCV NS4A changes the distribution of mitochondria in cells and causes mitochondrial damage, which finally induces apoptosis by activating caspase-3 (Nomura-Takigawa et al., 2006). Previous studies have reported that SVV 3C^pro^ induced apoptosis depending on its protease activity, which did not directly cleave or interact with PARP1 (Tingting et al., 2019). Similarly, our study showed that PSV 3C^pro^-induced PARP1 cleavage disappeared in the presence of Z-VAD-FMK.

Previous studies have shown that some picornaviruses could cleave MAVS at different sites. CVB3 and SVV 3C^pro^ induce the cleavage of MAVS at Gly148 (Mukherjee et al., 2011; Qian et al., 2017). EV71 2A^pro^ cleaves MAVS at multiple distinct sites (Bei et al., 2013). MAVS is cleaved during HRV1a infection by viral proteinases 2A^pro^, 3C^pro^, and activated caspase-3 (Drahos and Racaniello, 2009). Caspase-3 cleaves MAVS at D429/490 during virus infection and cell apoptosis (Ning et al., 2019); a series of MAVS mutants were constructed in which potential D residues were replaced with A residues. Similar to the previously reported study (Ning et al., 2019), we found that the key site of 3C^pro^-mediated MAVS cleavage was D429, and the cleavage fragments lost the ability to induce the production of type I IFN. Moreover, our data demonstrated that MAVS D429A still induced IFN-β production.

In summary, our results identify that PSV blocks host antiviral innate immunity by cleaving MAVS and degrading MDA5 and TBK1. The comprehensive mechanism of PSV suppressing the host innate immune response needs to be further explored.

## Data Availability Statement

The original contributions presented in the study are included in the article/supplementary material. Further inquiries can be directed to the corresponding authors.

## Author Contributions

WW drafted the main manuscript and performed the data analysis. WW, QZ and MY planned and performed the experiments. HW, XL, HC, QZ, and PQ were responsible for the experimental design. All authors contributed to the article and approved the submitted version.

## Funding

This work was supported by grants from the National Program on Key Research Project of China (2021YFD1800304), the National Natural Science Foundation of China (31772749, 32072841), the Fundamental Research Funds for the Central Universities Grant 2662017PY108, and the Natural Science Foundation of Hubei Province (2019CFA010).

## Conflict of Interest

The authors declare that the research was conducted in the absence of any commercial or financial relationships that could be construed as a potential conflict of interest.

## Publisher’s Note

All claims expressed in this article are solely those of the authors and do not necessarily represent those of their affiliated organizations, or those of the publisher, the editors and the reviewers. Any product that may be evaluated in this article, or claim that may be made by its manufacturer, is not guaranteed or endorsed by the publisher.
